# Consideration of sex and gender differences in addiction medication response

**DOI:** 10.1186/s13293-022-00441-3

**Published:** 2022-06-27

**Authors:** Sherry A. McKee, Aimee L. McRae-Clark

**Affiliations:** 1 Yale School of Medicine, 2 Church St South, Suite 109, New Haven, CT 06519 USA; 2 Medical University of South Carolina, Charleston, USA

**Keywords:** Sex, Gender, Addiction, Substance, Medication development, Nicotine, Alcohol, Cannabis, Opioids

## Abstract

Substance use continues to contribute to significant morbidity and mortality in the United States, for both women and men, more so than another other preventable health condition. To reduce the public health burden attributable to substances, the National Institute on Drug Abuse and the National Institute on Alcohol Abuse and Alcoholism have identified that medication development for substance use disorder is a high priority research area. Furthermore, both Institutes have stated that research on sex and gender differences in substance use medication development is a critical area. The purpose of the current narrative review is to highlight how sex and gender have been considered (or not) in medication trials for substance use disorders to clarify and summarize what is known regarding sex and gender differences in efficacy and to provide direction to the field to advance medication development that is consistent with current NIH ‘sex as a biological variable’ (SABV) policy. To that end, we reviewed major classes of abused substances (nicotine, alcohol, cocaine, cannabis, opioids) demonstrating that, sex and gender have not been well-considered in addiction medication development research. However, when adequate data on sex and gender differences have been evaluated (i.e., in tobacco cessation), clinically significant differences in response have been identified between women and men. Across the other drugs of abuse reviewed, data also suggest sex and gender may be predictive of outcome for some agents, although the relatively low representation of women in clinical research samples limits making definitive conclusions. We recommend the incorporation of sex and gender into clinical care guidelines and improved access to publicly available sex-stratified data from medication development investigations.

## Highlights

- Substance use continues to contribute to significant morbidity and mortality in the United States.
- To reduce the public health burden attributable to substances, the National Institute on Drug Abuse and the National Institute on Alcohol Abuse and Alcoholism have identified that medication development for substance use disorder is a high priority research area.
- We reviewed major classes of abused substances (nicotine, alcohol, cocaine, cannabis, opioids) demonstrating that, sex and gender have not been well-considered in addiction medication development research.
- When adequate data on sex and gender differences have been evaluated (i.e., in tobacco cessation), clinically significant differences in response have been identified between women and men. However, these data are not at all considered in clinical care guidelines.
- We recommend improved access to publicly available sex-stratified data from medication development investigations, to inform clinical practice and to improve treatment provided to women with substance use disorders.

## Introduction

Substance use continues to contribute to significant morbidity and mortality in the United States, for both women and men, more so than another other preventable health condition. Substance use and non-medical prescription use is a leading cause of preventable morbidity [1]. Recent estimates find that drug use costs men an average of 1.4 years of reduced life expectancy and 0.7 years for women [2]. Both licit (alcohol and tobacco), and illicit drugs cost the U.S. economy $740 billion dollars per year due to lost work productivity, health care, and crime [3].

To reduce the public health burden attributable to substances, the National Institute on Drug Abuse and the National Institute on Alcohol Abuse and Alcoholism have identified that medication development for substance use disorder is a high priority research area. Furthermore, both Institutes have stated that research on sex and gender differences in substance use medication development is a critical area [4, 5]. However, it is unknown whether sex and gender has been adequately considered in the design, analysis, and reporting of clinical trial results for substance use disorders.

As of 1993, the NIH Revitalization Act stated that women were required to be included into Phase III NIH-funded clinical trials to “ensure that the trial is designed and carried out in a manner sufficient to provide for a valid analysis of whether the variables being studied in the trial affect women or members of minority groups, as the case may be, differently than other subjects in the trial” [6]. While the inclusion of women increased in clinical trials [7], the majority of NIH-funded studies failed to analyze outcomes by sex [8, 9].

The failure to account for sex and gender in the design and analysis of medication trials has significant implications regarding medication efficacy, dosage, and adverse events for both women and men. For example, the classic 1989 study demonstrating that low-dose aspirin reduced cardiovascular events in men [10] wasn’t replicated in women until 2005. Findings demonstrated that low-dose aspirin did not reduce cardiovascular events in women [11]. The failure to account for sex differences in the dosing for zolpidem led to severe driving accidents the morning following zolpidem administration [12]. In a recent review examining classes of drugs spanning organ systems, Zucker and Pendergast [13] identify that elevated blood concentrations of medications were linked to significant and negative adverse events for women.

The purpose of the current narrative review is to highlight how sex and gender has been considered (or not) in medication trials for substance use disorders to clarify and summarize what is known regarding sex and gender differences in efficacy and to provide direction to the field to advance medication development that is consistent with current NIH ‘sex as a biological variable’ (SABV) policy [14]. Included studies were identified through literature reviews of medication trials and key term searches (e.g., sex differences, gender, women, nicotine, alcohol, cocaine, cannabis, opioids). We limited our review of potential targets to those which have demonstrated some efficacy for addiction treatment. Differences between women and men can be identified as sociocultural (gender) and biological (sex) [14]. For the purpose of the present narrative review, we will use the term ‘sex and gender’ to acknowledge that findings regarding medication efficacy are driven by both sociocultural and biological factors. However, when summarizing prior findings, use of sex or gender terminology will be dependent on study-specific information. In general, preclinical studies are report on sex-differences and human studies report on gender differences (or male/female differences). In the following sections we review major classes of abused substances (nicotine, alcohol, cocaine, cannabis, opioids), highlighting what is known or not known, about sex and gender differences in response to Food and Drug Administration (FDA)-approved medications for addiction, as well as other medication targets which have been evaluated to treat substance use disorders.

## Nicotine

Tobacco use remains the leading cause of morbidity and mortality in the United States, resulting in 480,000 deaths per year [15] and costing the US economy $300 billion dollars annually [16]. While smoking rates have declined over the past 50 years, they have not declined as rapidly in women, and rates have equalized between women and men (15.6% vs. 12.0%) [17]. Smoking leads to extensive medical consequences and many of these risks are greater for women compared to men, even after equating for tobacco exposure. For example, female smokers are at a greater risk of lung cancer, chronic obstructive pulmonary disease, and coronary heart disease [18–22]. Women also experience significant sex-specific health risks, primarily associated with their reproductive health including altered menstrual function, infertility, ectopic pregnancy, earlier menopause, and cancer of the cervix [23].

In addition to health care disparities, women also face disparities with regards to quitting smoking. With a meta-analysis, summarizing 190 efficacy trials, effectiveness trials, prospective observational studies, and cross-sectional studies, we demonstrated that the preponderance of evidence indicated that women were less likely to quit smoking when compared to men [24]. We hypothesize that one key factor underlying the difficulty in quitting for women are sex and gender differences in medication response. Specifically, that commonly used medications for smoking cessation are less effective for women. In the following sections, we will review sex and gender differences in medication response to FDA-approved smoking cessation medications and will also review additional medication targets for smoking cessation that have demonstrated some preliminary efficacy, highlighting whether sex and gender differences have been examined, and available findings.

### FDA-approved medications for smoking cessation

*Nicotine Replacement Therapies (NRT)*. Nicotine replacement is available in various formulations including transdermal patch, gum, lozenge, inhalers, and nasal spray. As an agonist therapy, NRT is the most commonly used medication for tobacco dependence, which provides controlled doses of nicotine to minimize withdrawal symptoms and craving for cigarettes. There are few differences in efficacy across the various formulations, with meta-analytic results demonstrating a 60% increase (relative risk) in quitting at 6 months [25].

Perkins and Scott [26] found that transdermal nicotine was 40% more efficacious for men compared to women at 6-month post quit attempt. In a review of 42 placebo-controlled trials, we found that 22 studies examined outcomes by sex and gender, and when men and women differed, women had poorer outcomes than men [27]. Across the 42 studies, there were no available data by sex on medication compliance, adverse events, withdrawal, or craving. Neuroimaging studies find that there is a sex difference in the upregulation of nicotinic acetylcholine receptors (nAChR) following acute abstinence from nicotine, providing a neurochemical explanation for why men have a preferential response to NRT. nAChR availability was significantly higher in male smokers compared to male nonsmokers in striatum, cortex and cerebellum, but female smokers did not have higher nAChR availability than female nonsmokers in any region [28]. It appears that male smokers have active nAChRs allowing NRT to function, whereas for female smokers, nAChRs remain in a down-regulated state potentially reducing the efficacy of NRT.

*Bupropion*. Bupropion, first marketed as an anti-depressant, has some antagonist activity at nAChRs, and blocks reuptake of dopamine and norepinephrine. Overall, bupropion increases rates of quitting by 62% (relative risk) in clinical trial findings [29]. In a meta-analysis of 4421 smokers, Scharf and Shiffman [30] found that bupropion equally increased rates of quitting in women (odds ratio = 2.47), and men (odds ratio = 2.53). However, rates of quitting overall were 21% lower in women, regardless of treatment condition.

*Varenicline.* Varenicline, is a partial nicotinic agonist, targeting the alpha4beta2 nAChR receptor, thought to underlie nicotine-related reinforcement. Varenicline has been shown to reduce tobacco craving, withdrawal symptoms, and the reinforcing effects of smoking relative to placebo [31]. A Cochrane review determined that the pooled relative risk ratio for continuous or sustained abstinence at 6 months or longer for varenicline with standard dosing (2 mg/d) vs. placebo was 2.27 (95% CI = 2.02–2.55; *n* = 6166) [32]. The efficacy of varenicline by sex has been examined, with available findings primarily documenting no differences in outcomes [33–43]. However, these comparisons were post-hoc, and studies did not have sufficient power to examine sex and gender differences in efficacy. In a large meta-analytic investigation examining 10,641 smokers, comprising 98% of all available Phase II and Phase III data examining varenicline vs. placebo, we demonstrated sex differences [44]. Overall, we found that quit rates in the placebo arms were lower in women, but absolute rates of quitting were equal across men and women. Thus, women had a larger relative response to varenicline, particularly for the short-term (46% more efficacious at the end of treatment) and intermediate (34% more efficacious at the 6-month follow-up) outcomes. Varenicline was equally effective for women and men at the 1-year follow-up. To date, varenicline is the only medication to addresses the disparity in quit rates seen in women and balances their rates of quitting to be equal to that of men.

The mechanism by which varenicline improves quitting in women is unknown. One potential hypothesis is related to the metabolism of nicotine, as women are more likely to clear nicotine more quickly from their systems which is partially mediated by estrogen [45]. Clinical trial findings document that faster metabolizers have greater success in quitting when randomized to varenicline, and slower metabolizers have greater success when randomized to nicotine replacement [46].

*Comparing NRT, Bupropion, and Varenicline.* A Cochrane review comparing the efficacy of transdermal nicotine, bupropion, and varenicline found that varenicline was superior to transdermal nicotine and bupropion, and that nicotine and bupropion, while effective, did not differ from each other [47]. Even though this analysis comprised 101,804 smokers, sex-based analyses were not conducted. We conducted a replication of this analysis with 14,389 smokers for the purpose of conducting sex-based analyses [48]. For women, varenicline was found to be more efficacious than transdermal nicotine or bupropion, and that neither nicotine or bupropion increased quitting in women. For men, all three medications were found to be effective (odds ratio’s > 1), but that there were no statistical differences between them. We then replicated these sex-dependent findings with Phase IV data from the Current Population Survey (*n* = 7,906) comparing varenicline to transdermal nicotine patch [49]. For women, varenicline was superior to nicotine, whereas for men, the efficacy of varenicline and transdermal nicotine did not differ. Such findings provide a clear suggestion for tailoring medication choice for tobacco dependence based on sex. For women, varenicline should be considered as a first line medication, whereas the choice for men is less clear.

### Cholinergic targets

Cholinergic targets increase, decrease, modulate, or duplicate the action of the neurotransmitter acetylcholine, which is a primary facilitator of the parasympathetic nervous system. In addition to NRT and varenicline, nAChRs have been targeted for smoking cessation medication development including agonists (e.g., cytisine, dianicline, encenicline), antagonists (e.g., mecamylamine), and positive allosteric modulators (e.g., galantamine). Across these targets, there has been little consideration of potential sex and gender differences. Cytisine is a partial agonist of alpha4beta2 nAChRs, similar to the pharmacological action of varenicline. In addition, similar to varenicline, cytisine has demonstrated sex differences in medication response when compared to NRT. For women, cytisine increased rates of quitting for women, whereas for men, it was noninferior [50]. Some early work demonstrated that mecamylamine, a non-specific nicotinic antagonist, might be more effective for women [51], but later trials did not support the efficacy of mecamylamine for smoking cessation [52].

### Antidepressants

Various formulations of antidepressants have been investigated for smoking cessation. Smoking and depression are highly co-morbid, and depression contributes to smoking cessation failure [53]. We have found that depression is more strongly tied to smoking behavior for women than it is for men [54]. Given that depression is twice as common in women as it is in men [55], it is plausible to anticipate sex and gender differences in anti-depressant medication response for smoking cessation. Across studies examining antidepressants for smoking cessation, few have reported sex and gender differences. Fluoxetine (a serotonin reuptake inhibitor) vs. placebo, was found to actually increase smoking in men [56] but not women. Sex-dependent predictors of quitting have been found for fluoxetine, in the absence of positive findings for smoking cessation. Fluoxetine was found to reduce levels of depression prior to quitting in women, which was associated with greater rates of cessation [57]. Selegiline, a monoamine oxidase B inhibitor, was found to increase rates of cessation in women at the 1 year follow-up [58], although other studies of selegiline which examined sex failed to find any differences [59]. Among the tricyclic antidepressants, the majority of the research has focused on nortriptyline (which targets norepinephrine and dopamine). Two of the larger clinical trials of nortriptyline did examine gender as a moderating influence, with null findings [60, 61].

### Noradrenergic targets

Noradrenergic transmission is involved in stress-reactivity, drug reinforcement, and pre-frontal control of cognitive function [62]. The first evidence to suggest that the noradrenergic system may be an effective therapeutic target for smoking cessation came from studies investigating clonidine, a non-selective agonist of α2 receptors. A Cochrane review found that clonidine increased rates of smoking cessation by an odds ratio of 1.6 and reduced tobacco craving and other tobacco withdrawal symptoms including anxiety, irritability, tension, and hunger [63, 64]. In studies that stratified by gender, clonidine was found to be more effective in women than in men [65–67]. In a meta-analysis (*n* = 813), end of treatment quit rates in women were 70% for clonidine vs. 18% for placebo. Men did not demonstrate an effect of medication with end of treatment quit rates of 42% for clonidine vs. 43% for placebo [68]. Surprisingly, mechanisms underlying this large sex difference in medication response were never pursued until recently.

We have been investigating guanfacine (an alpha2a agonist) for smoking cessation, hypothesizing that guanfacine may target sex-dependent mechanisms involved in smoking cessation, namely, stress. A substantial body of preclinical literature indicates that stress exposure induces relapse to drug seeking, including nicotine [69], a phenomenon mediated by brain stress circuits, including noradrenergic pathways [70]. Stress regulation plays an especially critical role in the maintenance of and relapse to smoking in women [71, 72]. With a small sample, we demonstrated that guanfacine attenuates the effect of stress on smoking behavior and increases quitting behavior [73]. With a larger sample, powered to examine the effect of sex on outcomes, we have demonstrated that guanfacine attenuates the effect of stress on precipitating smoking in women only [74]. Clinical trial work examining the efficacy of guanfacine for smoking cessation in women and men is ongoing (McKee; NCT04198116). We have also examined doxazosin (an alpha1 antagonist) and carvedilol (a combined alpha1 and beta antagonist) for smoking cessation, finding efficacy in the former, and no sex differences across either [75, 76].

### Antileptics

Antileptics (or anticonvulsants) are a diverse class of drugs often targeting GABA, glutamate, sodium channels, or calcium channels. Within this class of targets, topiramate, zonisamide, gabapentin, and pregabalin have been examined for smoking cessation, with mixed results [77–79]. With regards to sex differences, an early study by Anthenelli et al. [80] found striking differences in the efficacy of topiramate for smoking cessation. Overall, rates of abstinence with topiramate (16.3%) vs. placebo (15.9%) did not differ; however, when stratified by sex, rates of quitting with topiramate in men (37.5%) were substantially greater than in women (3.7%), an odds of almost 16 times. In fact, topiramate appeared to worsen outcomes for women. The authors note that adverse events unlikely accounted for the differences seen across men and women and suggest that sex differences in GABAergic tone in the mesocorticolimbic dopamine circuit [81] may account for their findings.

### Summary

There has been a long-standing acknowledgement of sex and gender differences in tobacco and nicotine use, which has translated to increased knowledge regarding sex and gender differences medication effects and associated mechanisms. While the majority clinical trials were not prospectively designed to consider sex and gender differences, women were recruited in sufficient samples to be able to discern sex and gender differences in efficacy of the FDA-approved medications. Across other promising medication targets, sex and gender differences are noted, with noradrenergic agents demonstrating promise in targeting stress-related smoking.

## Alcohol

Alcohol consumption is the third leading cause of preventable morbidity and mortality in the U.S. [82] and losses to the economy exceed $249 billion dollars per year [83]. Alcohol use disorder (AUD) is particularly problematic for the U.S., as we exceed global per capita alcohol consumption by 50% [84]. Historically, rates of AUD have been greater in men when compared to women; however, this gap is closing [85–89]. Over the past 10 years, rates of AUDs have increased in women by 84%, relative to a 35% increase in men [90].

While drinking is strongly associated with significant health risks in both sexes, females have exacerbated alcohol-related health consequences when compared to males. Women with AUDs have a higher risk of developing alcohol-related liver injury, including liver cirrhosis, hepatitis, and inflammation compared to men [91], even though women may consume less alcohol and have a shorter duration of use [92, 93]. Alcohol use increases the risk of cancers of the mouth, esophagus, pharynx, larynx, liver, and breast and women are at greater risk of alcohol-related cancer than men [91]. Women are more vulnerable to alcohol-related cardiovascular conditions than men. AUDs in women are associated with increased risk of cardiomyopathy [94], hypertension [95], and atrial fibrillation [96]. While there is some evidence that low to moderate alcohol consumption protects against ischemic stroke, there is an increased risk of stroke among women consuming 3 or more drinks per day relative to men [97].

In addition to experiencing greater relative risk than men for the most common and serious alcohol-related diseases, women also face sex-specific health consequences from drinking. Excessive alcohol consumption is associated with menstrual irregularity and altered hormone levels during menstrual cycle phases [98], including increases in endogenous estradiol [99] and temporary increases in testosterone levels [100]. These hormonal increases have been associated with incidence of female-related health problems, including cancers (e.g., breast) and poor reproductive health. Pregnancy- and perinatal-related consequences of drinking include infertility, spontaneous abortion [101], and perinatal mortality [102], as well as significant cognitive, psychological, and behavioral problems in offspring, including fetal alcohol syndrome [103]. Alcohol use is also associated with increased risk of physical and sexual assaults among women, with approximately one half of cases involving alcohol consumption by the either the victim, perpetrator, or both [104].

Abstinence from alcohol or the reduction of high-risk drinking can prevent and reduce many of the harmful health consequences of drinking. Successful treatment of AUD is associated with lowered blood pressure, improved liver function, and stabilization of many conditions, including cardiomyopathy, gastritis, ascites, and edema [105]. However, medication development for AUD has generally not considered potential sex and gender differences.

### FDA-approved medications for alcohol use

There are three FDA-approved medications for AUD: disulfiram, naltrexone (oral and depot formulations), and acamprosate. Disulfiram was first approved in 1948 for AUD, which inhibits the breakdown of alcohol and results in a toxic reaction to alcohol (e.g., nausea, vomiting). Disulfiram demonstrates some improvement in short-term abstinence [106]; however, this research was primarily conducted in males and there is insufficient data to determine if there are sex differences in treatment response [94]. A meta-analytic study found that women only accounted for 1% of all study participants evaluating the efficacy of disulfiram [107], negating any possible sex-based analysis.

Naltrexone, an opioid receptor antagonist, is hypothesized to attenuate alcohol craving with effects mediated through the mesolimbic dopamine (DA) system. Naltrexone has been found to reduce craving and drinking with generally small-to-medium size effects [106, 108–110]. While no studies have been designed to specifically address potential gender differences in naltrexone efficacy, post-hoc meta-analytic findings indicate that naltrexone may be equally effective for women and men [108–110]. However, women report greater adverse events in response to naltrexone, including nausea and sleep disturbances [110], and are more likely to discontinue treatment because of adverse events [111].

Acamprosate has a chemical structure similar to GABA and is thought to normalize the dysregulation of NMDA-mediated glutamatergic neurotransmission associated with chronic drinking. Evaluation of sex differences in medication response suggests that acamprosate may be equally effective for women [107]. In a meta-analytic study examining 1317 women and 4794 men, no gender differences were observed regarding acamprosate’s efficacy, safety, or tolerability [112].

### Cholinergic targets

The nicotinic partial agonist varenicline has been investigated for alcohol use [113], with mixed efficacy in clinical trials and laboratory studies [114]. In a recent meta-analysis summarizing findings from 10 studies, only 1 study reported data separately for men and women. In a sample of participants with alcohol use disorders who were co-morbid smokers, larger decreases in drinking were observed in men, vs. the smaller decreases observed in women [115]. In addition, men had higher rates of ‘no heavy drinking’ with varenicline vs. placebo than women [116].

### Noradrenergic targets

Alpha1 noradrenergic antagonists, both prazosin and doxazosin, have been tested in randomized clinical trials for AUD with mixed findings on drinking behavior. Studies to date have included low percentages of women [117] precluding the option of examining sex differences, or have covaried for sex in analyses [118, 119]. Only one study examining doxazosin had an a-priori hypothesis to examine sex differences in their data, with findings demonstrating no sex differences [120].

### Baclofen

Baclofen, a GABA_B_ agonist, demonstrated promise for the treatment of AUD in several small studies but clinical trial evidence has been mixed. Interestingly, studies are often designed with lower doses of baclofen for women but then, do not analyze for sex differences [121]. In a study designed to examine predictors of baclofen response [122], sex was not included as a predictor and the number of women included in the study was not reported. To date, two studies have addressed sex. Garbutt et al. [123] recruited a sample (*n* = 80) that was 45% women and examined sex as a moderator of medication effects with non-significant findings. In a larger RCT (*n* = 320), Reynauld et al. [124] found no overall efficacy for baclofen on abstinence; however, in stratified analysis, women administered baclofen were almost 11× more likely to achieve abstinence then men. Curiously, these differences were suggested to be due to longer detoxification periods in women, even though the effect of longer detoxification periods increased the odds of abstinence by only 6×. The authors do not discuss their significant sex finding.

### Ondansetron

Ondansetron is a selective serotonin 5-HT3 receptor antagonist demonstrating some positive findings in clinical trials for AUD. Initial studies found that moderating effects of early vs. late onset AUD on medication responses [125], and many subsequent trials have focused on moderating effects of genetic polymorphisms potentially related to the timing of AUD onset. In one such study, Kenna et al. [126] found that genetic polymorphisms interacted with sex on medication response. Women, but not men, who had LL genotype and equal or greater than 7 exon III repeats on dopamine receptor D4 gene, had significantly reduced alcohol intake when taking ondansetron.

### Other targets

A number of other targets have shown promise for the treatment of alcohol use disorder including anti-epileptics (topiramate, zonisamide, gabapentin), ABT-436 [127], aripiprazole [128], ghrelin [129], and glucocorticoids [130, 131]. However, none of these studies address potential sex and gender differences.

### Summary

Recent surges in rates of AUD in women, combined with exacerbated health risks in women due to alcohol consumption, identify that medication development needs to be responsive to known sex and gender differences in AUD. Across studies, there was reasonable consideration of well-known sex differences metabolism when setting drinking limits for eligibility criteria or trial endpoints (e.g., heavy drinking defined as 4+ drinks per episode for women and 5+ drinks per episode for men). Overall, women were recruited in low numbers, with no study recruiting 50% women and at best, percentages of women included in studies reflected population rates of AUD. A very small number of studies examined potential moderating effects of sex and gender, and fewer still included analysis of sex and gender differences in a-priori hypothesis. To date, there are two medications, naltrexone and acamprosate, with sufficient data to investigate potential sex differences in overall efficacy and adverse events.

## Cocaine

Cocaine use disorder is associated with a multitude of medical, psychiatric, and psychosocial consequences. Of the over 4 million past-year cocaine users in the United States, men represent the majority (60%) [132]. Despite this lower prevalence of use, there is evidence that women may actually have an increased vulnerability to the development and deleterious consequences of cocaine use. Several studies have found that women meet criteria for cocaine use disorder more quickly and enter treatment programs earlier as compared to men [133–135] but not all [136]. Women with a history of cocaine use disorder also have greater psychiatric, medical, social/family, and employment problems [137–140] and are more likely to attribute relapse to negative emotional states and interpersonal conflict than men [141, 142]. Furthermore, evidence suggests sex- and gender-specific alterations in neuroendocrine and HPA-axis responsivity [143–145], and cue- and stress-induced craving in individuals with cocaine use disorder [146]. In particular, the HPA axis and noradrenergic systems in women may be more sensitive to disruption resulting from chronic cocaine use and trauma exposure. These findings suggest sex and gender differences in cocaine use disorder which may have important treatment implications.

Current treatment approaches for cocaine use disorder typically are based on behavioral interventions. Although sex and gender analyses have not consistently been reported in behavioral treatment studies, when these data are included few differences in cocaine outcomes have been reported [147]. To date, there are no FDA-approved pharmacologic agents for cocaine use disorder, although cocaine use disorder medication development is an area of active research. In contrast to behavioral treatments, there is some suggestion of sex differences in clinical outcomes in trials of medications for cocaine use disorders when these factors have been examined.

### Disulfiram

Disulfiram, an acetylaldehyde dehydrogenase inhibitor, is FDA-approved for the treatment of alcohol use disorders, as discussed above. Proposed mechanisms of action of disulfiram for cocaine use disorder include reduction in comorbid alcohol consumption, impacts on cocaine metabolism, and normalization of dopaminergic tone [148]. Multiple studies have evaluated disulfiram combined with behavioral platforms for cocaine use disorder. An aggregate analysis of five trials (*n* = 434) found that women had worse treatment outcomes than men, which was primarily accounted for by disulfiram being less effective in women than men as gender differences were not observed among participants receiving behavioral treatment without disulfiram [147].

### Naltrexone

Naltrexone, FDA-approved for alcohol and opioid use disorders, has also been evaluated in cocaine use disorder for its potential effects on concomitant drinking behavior and cocaine craving [149–152] with limited efficacy. Pettinati et al. [152] stratified treatment assignment by gender; naltrexone reduced cocaine and alcohol use in men but not women. Furthermore, women randomized to naltrexone used more cocaine than women receiving placebo.

### Dopaminergic agents

Numerous dopamine agonists, such as long-acting dextroamphetamine and mixed amphetamine salts, have been evaluated as treatments for cocaine use disorder, based on promising agonist treatment outcomes in other addictive disorders [153]. Although some positive cocaine use outcomes have been reported using agonist interventions, outcomes by sex have generally not been included. A trial of modafinil reported improved outcomes in men receiving medication compared to placebo; a similar pattern was not observed in women [154].

### Noradrenergic targets

There is growing research to assess whether chronic cocaine-related adaptations in the noradrenergic system can be reversed by decreasing norepinephrine centrally and whether such interventions can attenuate withdrawal, neurocognitive dysfunction, and drug use. Guanfacine, an α2-receptor agonist, is an antihypertensive agent which reduces noradrenergic tone. Fox et al. [155] demonstrated in a human laboratory study that administration of guanfacine (up to 3 mg daily) reduced cocaine cue-induced craving, anxiety, and arousal. A follow-up trial evaluated the impact of 3 weeks of guanfacine treatment on stress- and cue-response in forty early abstinent cocaine-dependent individuals [156]. Guanfacine treatment attenuated stress- and cue-induced craving, anxiety, and negative emotion in women, but not in men.

### Other agents

Numerous other agents have been evaluated for treatment of cocaine use disorder, including topiramate [157, 158], bupropion [159] and cholinesterase inhibitors [160]. Recent research has also focused on targeting the stress pathway, using an 11-beta-hydoxylase inhibitor combined with a GABA-a positive allosteric modulator [161]. To date, however, potential sex and gender differences in response have not been addressed.

### Summary

Although significant effort has been made to find an efficacious pharmacotherapy for cocaine use disorder, no medication has shown significant efficacy to date. However, sex-disaggregated results have not been consistently reported. Of note, when analyses by sex have been conducted, women overall appear to have worse cocaine use outcomes than men in medication trials, although this pattern has generally not been seen with behavioral treatment studies.

## Cannabis

Cannabis use prevalence estimates show increasing use in recent years, likely due to changes in legalization and societal attitudes related to cannabis [162, 163]. Among people aged 12 or older in the United States, the percentage of past year cannabis users increased from 11.0 percent (or 25.8 million people) in 2002 to 17.5 percent (or 48.2 million people) in 2019 [132]. Prevalence of cannabis use has increased significantly among both pregnant and non-pregnant women [164, 165], with current estimates of 42.4% U.S. women endorsing lifetime cannabis use.

As occurs in other substances of abuse, men are at greater risk for development of lifetime cannabis use disorder (CUD) than women [166]. However, cannabis using women show greater abuse liability [167], more rapid progression from use to disorder [168, 169], more severe withdrawal symptoms [170–172], and greater barriers to care [173]. There are also concerns related to prenatal exposure to cannabis, and potential for poor offspring outcomes, such as low birth weight and impaired neurodevelopment [174].

Although a critical need for effective interventions exists, few specific treatments have been developed for CUD. To date, there are no FDA-approved medications for CUD, although multiple pharmacologic interventions have been evaluated. In the following sections, the data for specific CUD medication development target for which randomized, controlled outpatient trials have been conducted and considerations of sex in study design and data interpretation will be discussed.

### Antidepressants and anxiolytics

Given the high comorbidity of cannabis use with depression and anxiety, as well as overlap of cannabis withdrawal and affective symptomatology, multiple trials have evaluated antidepressants and anxiolytics for CUD with mostly negative results. The majority of these trials did not include sex in the outcome statistical analysis nor were findings reported stratified by sex [175–178]. Of note, when sex was considered, potentially clinically relevant differences in response were noted. A trial of the anxiolytic buspirone found no difference in cannabis use outcomes with buspirone or placebo treatment; however, women randomized to buspirone treatment had significantly worse outcomes than men randomized to buspirone [179]. In a study of the antidepressant vilazodone, women were found to have worse cannabis use outcomes compared to men [180]. Among a sample of adolescents with comorbid depression and CUD, Cornelius and colleagues [181] found no overall treatment effect of fluoxetine. However, the authors did observe a time by gender interaction, with females demonstrating greater improvement on depressive and cannabis-related symptoms than males.

### Endocannabinoid targets

The endocannabinoid system (ECS) is responsible for a wide array of internal processes associated with cannabis use and CUD. In addition to dopaminergic interactions, the ECS also appears to have a role in maintaining homeostasis, such as emotional regulation, sleep, feeding, and modulation of the stress response, and as such potentially contributes to cannabis withdrawal [182–187]. Given its multitude of effects, normalizing ECS signaling has been identified as a potential therapeutic target for CUD. Of note, the ECS is sexually dimorphic, and these sex differences may contribute to observed differences between men and women in the sociologic presentation of CUD and in presentation of cannabis withdrawal symptoms.

Dronabinol, an orally bioavailable formulation of delta-9-tetrahydrocannabinol (THC) and direct CB1 agonist, has been demonstrated to attenuate cannabis withdrawal symptoms in both inpatient and outpatient laboratory settings [188, 189]; however, two outpatient clinical trials have not shown dronabinol to be effective for promotion of cannabis abstinence either alone or in combination with the adrenergic agonist lofexidine [190, 191]. Sex differences in outcomes were not examined or reported in either trial.

Cannabidiol, although similar in structure to THC, binds poorly to CB1 and CB2 [192]. However, cannabidiol exerts effects within the ECS, acting as a negative allosteric modulator of the CB1 receptor and inhibiting reuptake and hydrolysis of the endogenous cannabinoid ligand anandamide [193, 194]. A recent clinical trial by Freeman et al. [195] demonstrated modest improvement in urinary cannabinoid reductions and days abstinent with cannabidiol treatment compared to placebo. Post-hoc analyses examining sex differences did not change outcomes.

Nabiximols is an oromucosal spray composed of THC (2.7 mg/spray), cannabidiol (2.5 mg/spray), and various terpenoids. Two randomized clinical trials have evaluated nabiximols as a potential treatment for CUD. One trial failed to find significant differences in cannabis withdrawal or cannabis [196]. A larger recent trial found a reduction in self-reported cannabis using days among individuals receiving nabiximols relative to placebo [197]. The impact of sex on outcomes was not assessed or reported in either trial.

The ECS also presents targets for CUD in the form of biosynthetic and degradative enzymes, for which activity can be either facilitated or inhibited to indirectly modulate endogenous cannabinoid levels. One trial has been completed thus far evaluating the FAAH-inhibitor PF-0447845, showing attenuate cannabis withdrawal symptoms and self-reported cannabis use [198]. However, the sample only included men, precluding any comparison of outcomes by sex.

### Other targets

*N*-Acetylcysteine (NAC), an *N*-acetyl pro-drug of the naturally occurring amino acid cysteine, has been shown to increase non-synaptic glial release of glutamate via stimulation of the cystine–glutamate exchange which becomes dysregulated after chronic drug use. A placebo-controlled study showed that NAC, when paired with contingency management (CM) to promote abstinence, doubled the odds of negative urine cannabinoid tests during treatment among cannabis use disordered adolescents [199]; however, a large multisite study of adults did not find any differences on cannabis use outcomes with NAC treatment [200]. In both studies, the effect of sex on outcomes was examined, with no impact of sex observed. Other medications that have been evaluated for CUD in randomized trials include the anticonvulsants divalproex [201], gabapentin [202], and topiramate [203] as well as the attention deficit hyperactivity medication atomoxetine [204]. No improvement in cannabis abstinence was found in any of these trials nor was sex included in outcome analyses.

### Summary

Despite growing evidence demonstrating important sex and gender differences in course and sequelae of CUD, of the nineteen randomized, outpatient trials for CUD conducted to date only six reported any sex-disaggregated outcomes. However, half of the trials that did include sex-specific analyses found an impact of sex either on medication effect or treatment outcome generally. Of these three, two studies reported worse outcomes with the pharmacologic intervention in women relative to men [179, 180], suggesting that, as with nicotine use disorder, gender may need to be a consideration in medication selection. This may be particularly relevant for agents targeting the ECS, given identified differences in endocannabinoid signaling, as well as adrenergic targets given the gender differences noted in medication response in other use disorders. One potential obstacle in appropriately assessing gender differences is inclusion of a sufficient sample of females in clinical trials. In the CUD trials discussed above, less than 25 percent of participants were female. Although historically men have used cannabis more often than women, this gender gap has narrowed in recent years [205, 206], underscoring the need for greater inclusion of females in future research and prospective considerations of sex and gender in study design.

## Opioids

Opioid use disorder (OUD) is increasing at alarming rates in the United States. While the number of opioid-dependent men in the US remains larger than the number of opioid-dependent women, there are some disturbing trends. Since 1999, prescription opioid overdose deaths in the United States increased 642 percent in women compared to an increase of 439 percent in men [207]. Among U.S. women 30 to 64 years of age, severe increases have occurred between 1999 and 2017 in deaths involving synthetic opioids (1643%) and heroin (915%) [208]. During this time period, the average age for drug overdose deaths increased by 3 years for women in this age group, suggesting that middle-aged women may be in particular need of treatment interventions [209].

### Opioid replacement therapy

Opioid replacement therapy is the standard of care for OUD, with evidence showing significant reduction in illicit drug use, including opioid use, relapse, and death from OUD, and improvements in overall health [210]. Opioid agonist treatment includes administration of a full agonist (methadone or diacetylmorphine) or partial opioid agonist (buprenorphine or buprenorphine/naloxone), whereas opioid antagonists (such as oral and extended-release naltrexone) block the euphoric and sedating effects of opioids to reduce craving and mitigate withdrawal symptoms. Few studies to date have been prospectively designed to assess sex differences in opioid replacement therapy outcomes [211, 212]. However, some evidence suggests that men women may respond differentially to opioid agonist agents. A study comparing methadone and diacetylmorphine found women receiving diacetylmorphine to be more likely to have reductions in illicit opioid use compared to women receiving methadone [213]. A trial comparing buprenorphine and methadone found that women receiving buprenorphine had greater treatment retention and less opioid use compared to men [214]. Similarly, Jones and colleagues [215] reported among men and women receiving either buprenorphine or methadone that women receiving buprenorphine were less likely to relapse than men receiving buprenorphine. Women receiving buprenorphine were also less likely to relapse compared to women receiving methadone.

### Noradrenergic agents

Alpha-2 agonists have been shown to ameliorate some signs and symptoms of opioid withdrawal [216]. Clonidine has long been used in opioid detoxification although it has not been FDA-approved for this indication. Lofexidine, also an alpha-2 agonist, became the first non-opioid medication FDA-approved for opioid withdrawal mitigation in 2018. Three studies and a Cochrane database systematic review comparing the efficacy and tolerability of lofexidine to clonidine suggest comparable efficacy in reducing withdrawal, with an improved risk–benefit profile for lofexidine including better tolerability and less hypotension [217–220]. Sex-disaggregated results were not reported for the lofexidine FDA-registration trials [221, 222]. Although traditionally used for opioid detoxification, there has been research interest in utilizing noradrenergic agents for opioid relapse prevention. Kowalczyk et al. [223] conducted a randomized double-blind, placebo-controlled relapse prevention trial of adjunctive clonidine treatment in 118 abstinent OUD men and women on buprenorphine. The clonidine-treated group had a significantly longer duration of abstinence as compared to the placebo group. Ecological monitoring assessment demonstrated that daily life stress was partly decoupled from opioid craving in the clonidine group, suggesting that clonidine may exert its beneficial effects by muting the stress response. The number of women in the sample was not large enough to support gender-specific data analysis. In a study of 18 opioid-dependent individuals stabilized on naltrexone, treatment with lofexidine vs. placebo was tested [224]. During the 4-week treatment period, the lofexidine patients had higher abstinence rates and improved relapse outcomes as compared to the placebo group. Ten subjects participated in a human laboratory stress/drug cue exposure paradigm. The lofexidine patients had significantly lower heart rate and attenuated stress and drug cue response as compared to the placebo group. However, as in the trial by Kowalczyk, the sample size did not support a gender-specific analysis in this study.

### Summary

To date, most studies of opioid replacement therapy have not included sex-specific analyses, which limit conclusive statements on sex and gender differences in response. Female representation in medication for opioid use trials has also historically been low, which further complicates data interpretation. In a recent systematic review comparing outcomes with buprenorphine to other opioid replacement interventions, Ling and colleagues [212] found that women represented only 26% of participants over 25 studies. Furthermore, small sample sizes have precluded examining sex differences in noradrenergic agent utility in OUD. Given the promising data from other use disorders as well as known sex influences in noradrenergic stress responding, it is important that any future trials of adrenergic agents in OUD are prospectively designed to assess for sex differences.

## Summary and future considerations

The current review demonstrates that, to date, sex and gender have not been well-considered in addiction medication development research. As summarized in Table 1, SABV has not been prospectively incorporated into study design, but has been included in post-hoc analysis which are often under-powered. When adequate data on sex and gender differences have been evaluated (i.e., in tobacco cessation), clinically significant differences in response have been identified between women and men. Across the other drugs of abuse reviewed, data also suggest that sex may be predictive of outcome for some agents, although the relatively low representation of women in clinical research samples limits making definitive conclusions.

**Table 1:** Summary of incorporation of sex as a biological variable into addiction medication study design, analysis, and efficacy outcomes (associated references in brackets)

Substance/target	SABV incorporated into any study design	SABV incorporated into any analysis	Sex difference in efficacy
Nicotine			
Nicotine replacement	No	Yes	*W* < M (26, 27)
Varenicline	No	Yes	*W* > *M* (31, 44, 48, 49)
Bupropion	No	Yes	*W* < *M* (30)
Cholinergic	No	Yes	*W* > *M* (cytisine) (50)
Antidepressants	No	Mixed	Mixed (56–61)
Noradrenergic	Yes	Yes	*W* > *M* (68, 74)
Antileptics	No	Yes (topiramate)	*W* < *M* (topiramate) (80)
Alcohol			
Naltrexone	No	Yes	*W* = *M* (108–110)
Acamprosate	No	Yes	*W* = *M* (112)
Disulfiram	No	No	Unknown
Cholinergic	No	Yes	*W* < *M* (115, 116)
Noradrenergic	No	Yes (doxazosin)	*W* = *M* (doxazosin) (120)
Baclofen	No	Yes	*W* > *M* (124)
Ondansetron	No	Yes	*W* > *M* (126)
Cocaine			
Disulfiram	No	Yes	*W* < *M* (147)
Naltrexone	No	Yes	*W* < *M* (152)
Dopaminergic	No	Yes (modafinil)	*W* < *M* (154)
Noradrenergic	Yes	Yes	*W* > *M* (156)
Cannabis			
Antidepressant/Anxiolytics	No	Mixed	Mixed (179, 180, 181)
Endocannabinoid targets	No	Yes (cannabidiol)	*W* = *M* (cannabidiol) (195)
*N*-Acetylcysteine	No	Yes	*W* = *M* (199, 200)
Opioids			
MAT	No	Mixed	*W* > *M* (buprenorphine) (214, 215)
Noradrenergic	No	No	No

While certain classes of targets (i.e., noradrenergic, cholinergic, antidepressants, GABA) have been studied across substances, the limited number of investigations which have evaluated sex differences makes conclusions across substances premature. To date, only noradrenergic targets appear to demonstrate consistent findings (with women having better outcomes) for tobacco and cocaine use (see Table 1). It is possible that targeting stress as a treatment strategy is particularly effective for women with addiction. When considering other treatment rationales (e.g., agonist therapy to treat withdrawal or antagonist therapy to block reinforcement), there does not appear to be complementary sex findings across substances with the limited studies currently available. Furthermore, co-use of substances commonly occurs (e.g., concomitant nicotine and alcohol or cannabis use), and some classes of drugs discussed above may have utility across substances. Although limited work to date has evaluated medications for co-occurring substance use disorders, sex and gender are critical to incorporate into these investigations.

In addition to efficacy outcomes, it is imperative to consider sex differences in adverse drug reactions in addiction medication development as these may impact medication compliance and retention in treatment. Women typically have a lower lean body mass and reduced hepatic clearance as compared to men, and there are also sex differences in cytochrome P450 activity impacting drug metabolism [225]. Sex differences in pharmacokinetics have been shown to strongly predict sex-specific adverse drug reactions for women but not for men [13]. However, most clinical trials reviewed did not consider sex differences in body weight and pharmacokinetics in medication dosing. Sex-disaggregated adverse events, retention, and compliance were also not typically reported.

Sex and gender should also be an important consideration in selecting future targets for addiction medication development. Across most substances of abuse, women demonstrate greater stress-related vulnerability, more severe withdrawal, and greater negative affect-related use/relapse. As such, translational research investigating these targets could be high yield for sex/gender-informed treatment development if efforts are made to include adequate samples of women.

## Recommendations

In this section we offer two recommendations to improve clinician and researcher access to gender-informed treatment recommendations and to sex-stratified data. The first recommendation concerns clinical care guidelines which have not well considered sex and gender differences. For example, the current clinical care guidelines for tobacco dependence, written in 2008, [226], state that “There is evidence that NRT can be effective with both sexes; however, evidence is mixed as to whether NRT is less effective in women than men. This may encourage the clinician to consider use of another type of medication with women, such as bupropion SR or varenicline”. The evidence that this statement is based upon is now dated. There is fairly strong evidence that NRT is less effective for women, and growing evidence that varenicline is relatively more effective for women vs. bupropion and NRT. Based on current available evidence, varenicline may be supported as a first line medication for women. Similarly, the American Psychiatric Association’s guidelines for alcohol pharmacotherapy is essentially silent on sex and gender differences, although they do have a section about treating pregnant women. These guidelines also acknowledge that increased research on sex and gender is needed to allow for personalized medication selection and tailored treatment plans. The American Society on Addiction Medicine national guideline for the treatment of opioid use disorder also addresses treatment of pregnant women but does not address potential differences in medication response or treatment considerations in non-pregnant women [227].

Our second recommendation requires that federal agencies report data by sex is critical to support the inclusion of sex into the design, analysis, and reporting of addiction medication development studies. For example, the FDA has been actively involved in supporting the study of sex differences in medication efficacy and adverse events. Since the 1998 Demographic Rule, new drug application content and “format regulations at 21 CFR 314.50 to require effectiveness data to be presented by gender, age and racial subgroups and dosage modifications be identified for specific subgroups. There is also a requirement that safety data be presented by gender, age and racial subgroups; and that safety data from other subgroups of the populations of patients treated be presented, as appropriate” [228]. While data are required to be presented by sex for New Drug Applications (NDAs), this information is rarely available as part of the prescribing information. We are advocating that FDA should provide that sex-stratified information, submitted as part of the NDA, be made publicly available. Based on the FDAAA 801 and the Final Rule [229], all clinical trial registration and results require posting on Clinicaltrials.gov. Currently, the only required sex-based information is the baseline percentage of women and men in clinical samples. To provide public access to sex-based data, it would be possible to require that the reporting of all investigational studies (efficacy and adverse events) be segregated by sex as part of Clincialtrials.gov reporting. Such transparency in and availability of clinical trial data is critical to the development of safe and effective medications for women with substance use disorders.

## Perspectives and significance

In this narrative review, we identify that sex and gender have not been well considered in medication develop for addiction. When adequate data on sex and gender differences have been evaluated (i.e., in tobacco cessation), clinically significant differences in response have been identified between women and men. We recommend that clinical care guidelines adopt sex and gender-specific recommendations when there are sufficient data to do so. We also recommend improved access to publicly available sex-stratified data from medication development investigations, to inform clinical practice and to improve treatment provided to women with substance use disorders.

## Data Availability

Not applicable.
