# Correction to: Consideration of sex and gender differences in addiction medication response

**DOI:** 10.1186/s13293-022-00449-9

**Published:** 2022-07-13

**Authors:** Sherry A. McKee, Aimee L. McRae-Clark

**Affiliations:** 1 Yale School of Medicine, 2 Church St South, Suite 109, New Haven, CT 06519 USA; 2 Medical University of South Carolina, Charleston, SC USA

## Correction to: Biology of Sex Diferences (2022) 13:34 10.1186/s13293-022-00441-3

Following publication of the original article [1], the authors reported a typo in the first sentence of the "Abstract" and the "Introduction". The first sentences of both should read as follows:

“Substance use continues to contribute to significant morbidity and mortality in the United States, for both women and men, more so than any other preventable health condition.”

The original article [1] has been corrected.
